# Supplementary material for: Unsupervised Hierarchical Clustering of Pancreatic Adenocarcinoma Dataset from TCGA Defines a Mucin Expression Profile that Impacts Overall Survival
Source: Cancers (Basel). 2020 Nov 9;12(11):3309. doi: 10.3390/cancers12113309 (PMC7697168; doi:10.3390/cancers12113309)
Supplement: Supplementary file 1 [file cancers-12-03309-s001.pdf]

Supplementary Materials

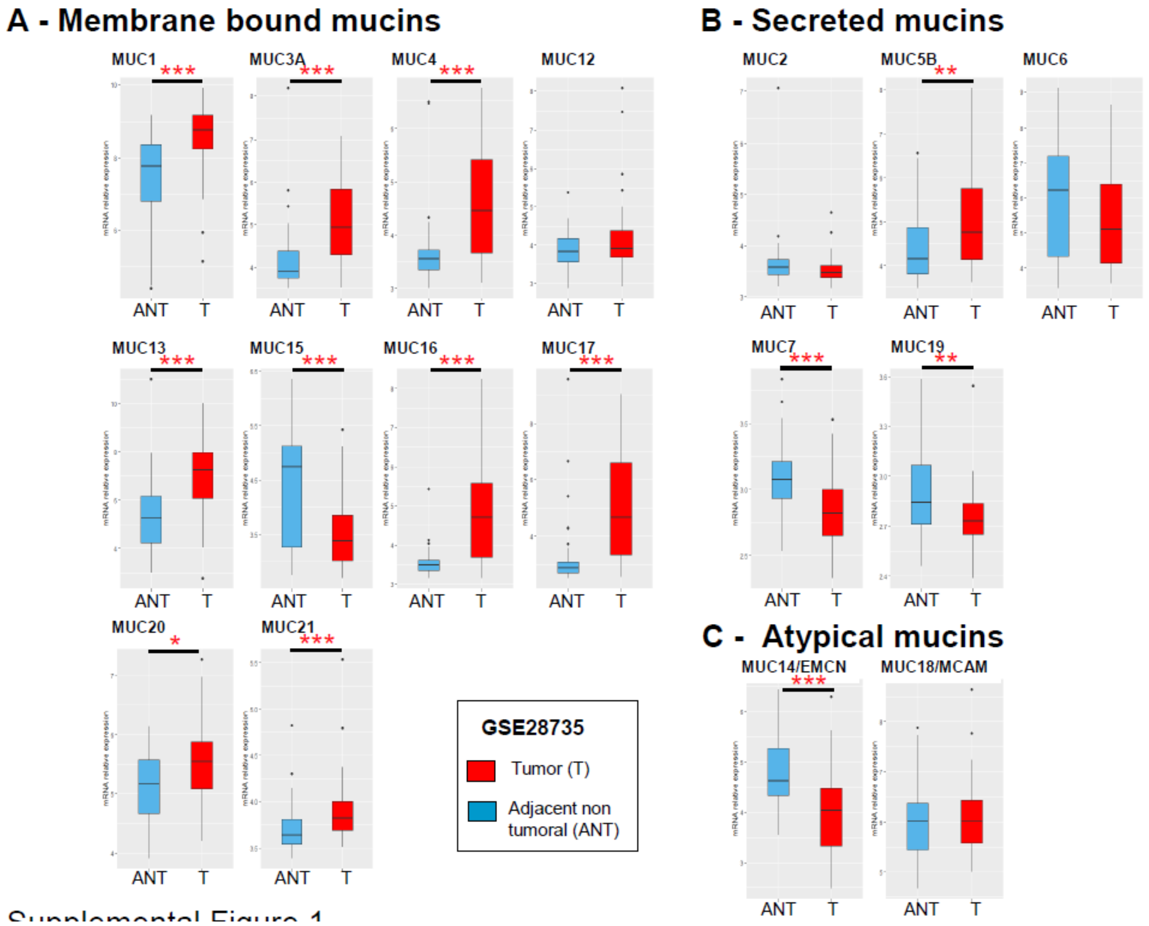

Figure S1. Mucin mRNA level in human tumor (T) and adjacent nontumor (ANT) tissues. (**A**) Membrane-bound mucin mRNA (*MUC1*, *MUC3A*, *MUC4*, *MUC12*, *MUC13*, *MUC15*, *MUC16*, *MUC17*, *MUC20*, and *MUC21*), secreted mucin mRNA (*MUC2*, *MUC5AC*, *MUC5B*, *MUC6*, *MUC7*, and *MUC19*), or atypical mucin mRNA (*MUC14/EMCN*, and *MUC18/MCAM*) expression was evaluated in PDAC d**atasets of the NCBI Gene Expression** Omnibus (GEO, GSE28735) database to analyze whether the mRNA level differed between adjacent nontumor tissues and tumor tissues. Statistical analyses were performed using paired *t*-test (*** $p < 0.001$, ** $p < 0.01$, * $p < 0.05$).

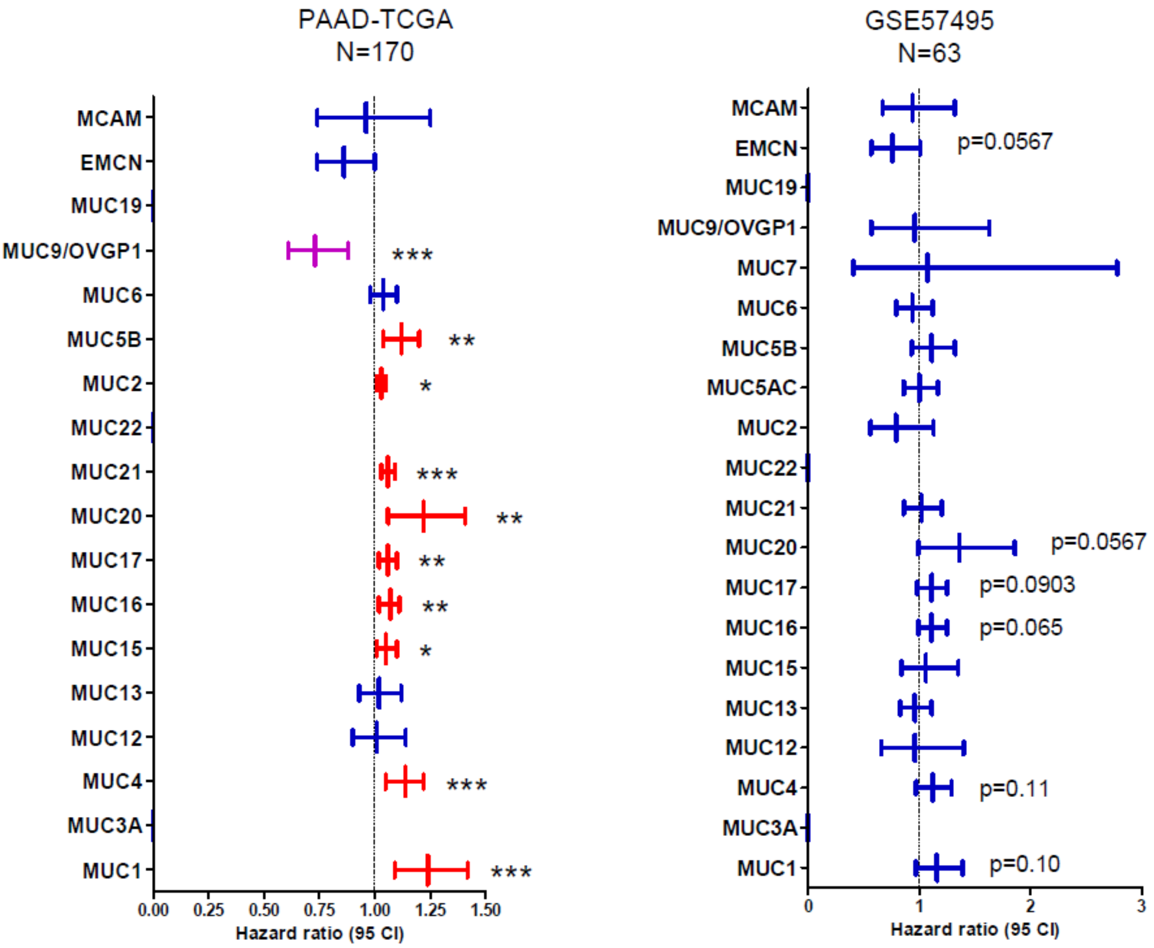

Figure S2. Mucin mRNA levels and hazard ratio in pancreatic adenocarcinoma PAAD and GSE57495 cohorts. Hazard ratio was evaluated using the PROGgeneV2 algorithm in PAAD-TCGA and GSE57495 datasets. Statistically significant hazard ratios ($p < 0.05$) are represented in red (HR > 1) or purple (HR < 1). The p-values are indicated on the graph (*** $p < 0.001$, ** $p < 0.01$, * $p < 0.05$).

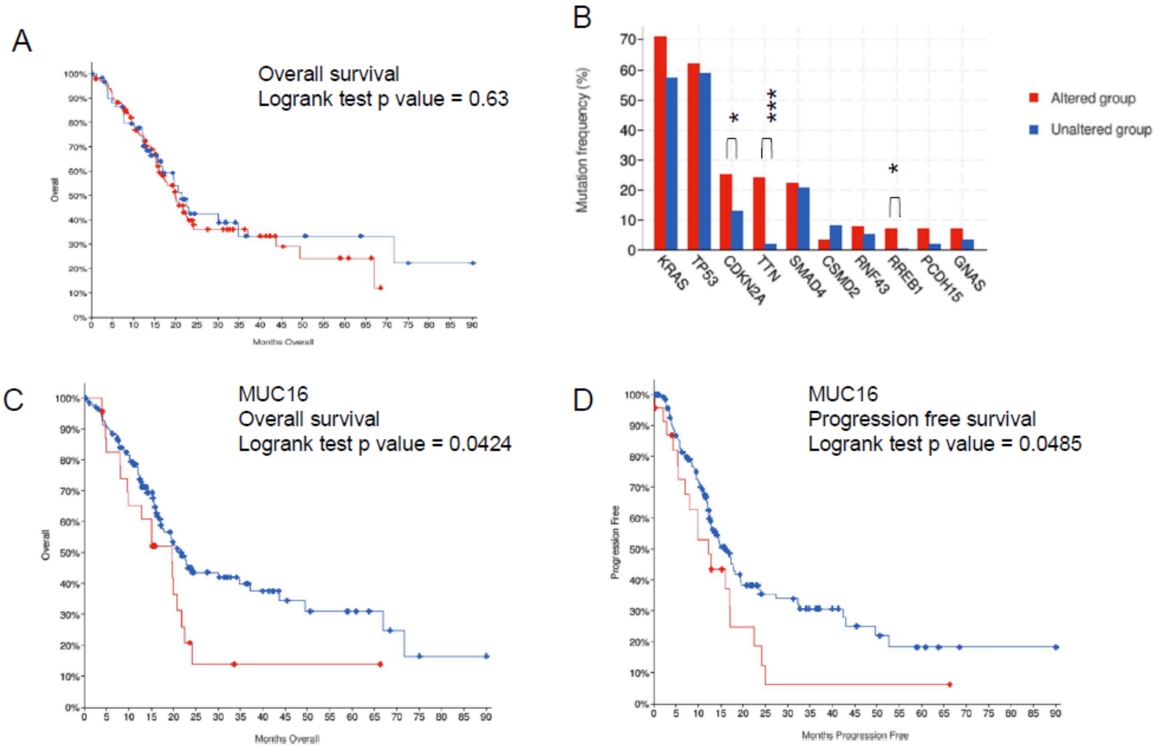

Figure S3. Analysis of overall survival and gene mutation in PAAD-TCGA patients with alterations of mucin genes. (**A**) Overall survival of PAAD-TCGA patients with at least one alteration event (amplification/deletion/high mRNA) of any mucin genes and nonaltered patients. (**B**) Comparison of most frequent gene mutation in PAAD-TCGA patients with at least one alteration of any mucin gene compared to nonaltered patients. Statistical analyses were performed using a paired *t*-test (*** $p < 0.001$, * $p < 0.05$). (**C**) Overall survival and (**D**) progression-free survival of PAAD-TCGA patients with at least one alteration event of MUC16 ($n = 23$) and nonaltered patients.

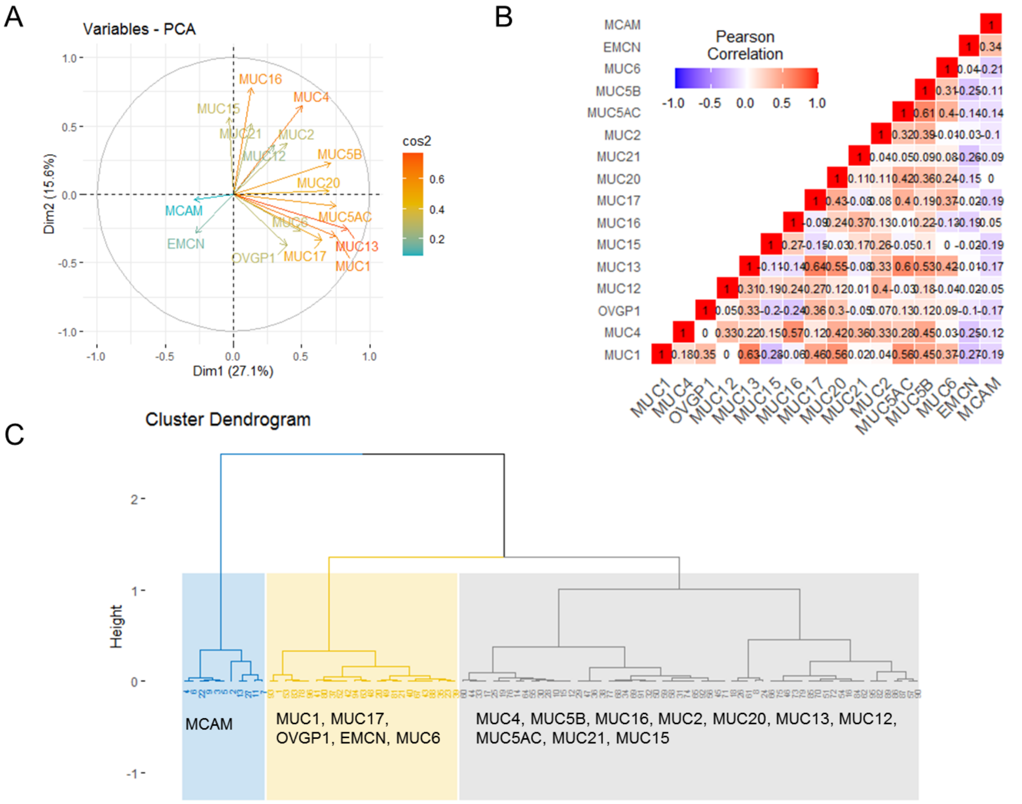

Figure S4: Correlation analysis of relative mucin mRNA levels in the independent QCMG pancreatic adenocarcinoma dataset. (**A**) Principal component analysis of mucin mRNA relative expression in QCMG cohort. (**B**) Correlation Pearson *r* values were calculated for each mucin mRNA combination from the QCMG cohort. (**C**) Unsupervised hierarchical clustering analysis of PDAC patients according to their relative mucin mRNA expression.

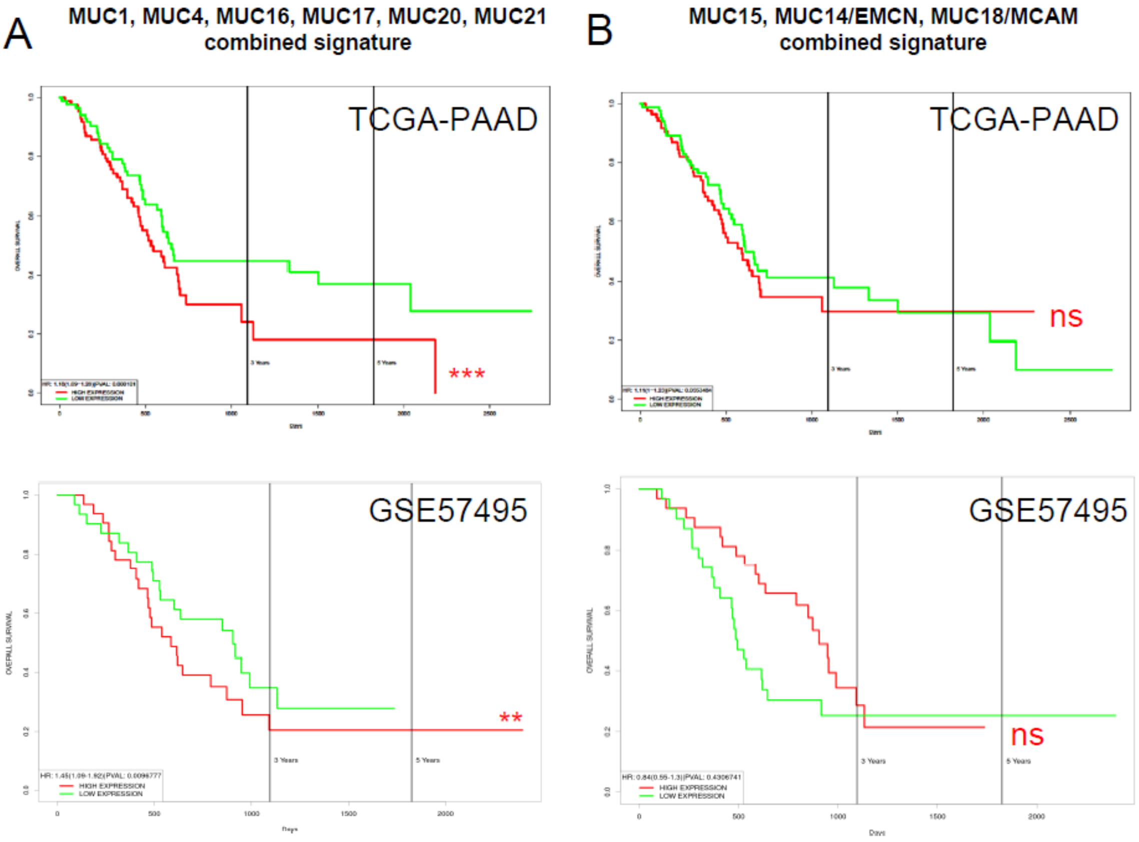

Figure S5. Analysis of overall survival of the #1 and #2 mucin signatures in pancreatic cancer datasets using PROGgeneV2. (**A**) Pancreatic cancer patients were stratified using a gene signature combining *MUC1*, *MUC4*, *MUC16*, *MUC17*, *MUC20*, and *MUC21*. Kaplan–Meier curves were analyzed with the PROGgeneV2 tool in the PAAD-TCGA and GSE57495 datasets. (**B**) PAAD patients were stratified using a gene signature combining *MUC15*, *MUC14/EMCN*, and *MUC18/MCAM* in PAAD-TCGA and GSE57495 datasets.

Table S1. Correlation of mucin gene expression in pancreatic adenocarcinoma QCMG dataset ($p < 0.05$). A total of 96 mRNA transcriptome samples were available.

Positive correlation			
**Gene A**	**Gene B**	**Pearson *r***	***p*-value**
MUC13	MUC17	0.64	0.00000
MUC1	MUC13	0.63	0.00000
MUC5AC	MUC5B	0.61	0.00000
MUC13	MUC5AC	0.60	0.00000
MUC4	MUC16	0.57	0.00000
MUC1	MUC20	0.56	0.00000
MUC1	MUC5AC	0.56	0.00000
MUC13	MUC20	0.55	0.00000
MUC13	MUC5B	0.53	0.00000
MUC1	MUC17	0.46	0.00000
MUC4	MUC5B	0.45	0.00000
MUC1	MUC5B	0.45	0.00001
MUC17	MUC20	0.43	0.00001
MUC4	MUC20	0.42	0.00002
MUC20	MUC5AC	0.42	0.00002
MUC13	MUC6	0.42	0.00002
MUC5AC	MUC6	0.40	0.00005
MUC12	MUC2	0.40	0.00006
MUC17	MUC5AC	0.40	0.00007
MUC2	MUC5B	0.39	0.00009
MUC16	MUC21	0.37	0.00019
MUC1	MUC6	0.37	0.00019
MUC17	MUC6	0.37	0.00022
MUC4	MUC21	0.36	0.00027
OVGP1	MUC17	0.36	0.00032
MUC20	MUC5B	0.36	0.00034
MUC1	OVGP1	0.35	0.00042
EMCN	MCAM	0.34	0.00069
OVGP1	MUC13	0.33	0.00094
MUC4	MUC12	0.33	0.00098
MUC4	MUC2	0.33	0.00104
MUC13	MUC2	0.33	0.00104
MUC2	MUC5AC	0.32	0.00165
MUC5B	MUC6	0.31	0.00180
MUC12	MUC13	0.31	0.00203
OVGP1	MUC20	0.30	0.00340
MUC4	MUC5AC	0.28	0.00623
MUC12	MUC17	0.27	0.00676
MUC15	MUC16	0.27	0.00774
MUC15	MUC2	0.26	0.01001
MUC16	MUC20	0.24	0.01868
MUC20	MUC6	0.24	0.01950
MUC12	MUC16	0.24	0.02001
MUC4	MUC13	0.22	0.03362
MUC16	MUC5B	0.22	0.03414
**Negative correlation**			
MUC1	MUC15	−0.28	0.00659
MUC1	EMCN	−0.27	0.00900
MUC21	EMCN	−0.26	0.00984
MUC4	EMCN	−0.25	0.01251
MUC5B	EMCN	−0.25	0.01599
OVGP1	MUC16	−0.24	0.01948
MUC6	MCAM	−0.21	0.04039
